# Socioeconomic determinants of access to health services among older adults: a systematic review

**DOI:** 10.1590/S1518-8787.2017051006661

**Published:** 2017-05-08

**Authors:** Ana Paula Santana Coelho Almeida, Bruno Pereira Nunes, Suele Manjourany Silva Duro, Luiz Augusto Facchini

**Affiliations:** I Departamento de Ciências da Saúde. Universidade Federal do Espírito Santo. São Mateus, ES, Brasil; II Programa de Pós-Graduação em Epidemiologia. Universidade Federal de Pelotas. Pelotas, RS, Brasil; III Departamento de Enfermagem. Universidade Federal de Pelotas. Pelotas, RS, Brasil; IV Departamento de Medicina Social. Faculdade de Medicina. Universidade Federal de Pelotas. Pelotas, RS, Brasil

**Keywords:** Aged, Health Services Accessibility, Socioeconomic Factors, Health Systems, Health Inequalities, Review, Idoso, Acesso aos Serviços de Saúde, Fatores Socioeconômicos, Sistemas de Saúde, Desigualdades em Saúde, Revisão

## Abstract

**OBJECTIVE:**

The objective of this study was to analyze the association between the socioeconomic characteristics and the access to or use of health services among older adults.

**METHODS:**

This is a systematic review of the literature. The search has been carried out in the databases PubMed, LILACS and Web of Science, without restriction of dates and languages; however we have included only articles published in Portuguese, English, and Spanish. The inclusion criteria were: observational design, socioeconomic factors as variables of interest in the analysis of the access to or use of health services among older adults, representative sample of the target population, adjustment for confounding factors, and no selection bias.

**RESULTS:**

We have found 5,096 articles after deleting duplicates and 36 of them have been selected for review after the process of reading and evaluating the inclusion criteria. Higher income and education have been associated with the use and access to medical appointments in developing countries and some developed countries. The same association has been observed in dental appointments in all countries. Most studies have shown no association between socioeconomic characteristics and the use of inpatient and emergency services. We have identified greater use of home visits in lower-income individuals, with the exception of the United States.

**CONCLUSIONS:**

We have observed an unequal access to or use of health services in most countries, varying according to the type of service used. The expansion of the health care coverage is necessary to reduce this unequal access generated by social inequities.

## RESUMO

**OBJETIVO:**

Analisar a associação entre características socioeconômicas e acesso ou utilização de serviços de saúde entre idosos.

**MÉTODOS:**

Revisão sistemática da literatura. A busca foi realizada nas bases de dados PubMed, Lilacs e Web of Science, sem restrição de datas e idiomas, entretanto foram incluídos somente os artigos publicados em português, inglês e espanhol. Foram critérios de inclusão: ter delineamento observacional; possuir os fatores socioeconômicos como variáveis de interesse na análise do acesso ou utilização de serviços de saúde entre idosos; ter amostra representativa da população alvo; fazer ajuste para fatores de confusão; e não apresentar viés de seleção.

**RESULTADOS:**

Foram encontrados 5.096 artigos após a exclusão de duplicidades e 36 foram selecionados para a revisão após o processo de leitura e avaliação dos critérios de inclusão. Maior renda e escolaridade estiveram associadas à utilização e acesso a consultas médicas nos países em desenvolvimento e em alguns países desenvolvidos. A mesma associação foi observada nas consultas odontológicas em todos os países. A maioria dos estudos não apresentou associação entre características socioeconômicas e uso de serviços de internação e emergência. Foi identificado maior uso de visita domiciliar em indivíduos de menor renda, com exceção dos Estados Unidos.

**CONCLUSÕES:**

Observou-se desigualdade no acesso ou na utilização de serviços de saúde na maior parte dos países, variando em relação ao tipo de serviço utilizado. A ampliação da cobertura de serviços de saúde faz-se necessária para a redução da desigualdade no acesso gerada por iniquidades sociais.

## INTRODUCTION

Access to health services is one of the main factors in the analysis of the quality and performance of health systems^14,58^. Access is a set of dimensions that describe the adjustment between the individual and the health care system, i.e., it intermediates the relationship between demand and entry in the service^43^. The use of health services comprises all direct contact with these services and can be understood as the evidence that access has been reached^55^. Although related, the access to and the use of health services are not synonymous, as seem in much of the literature.

The older adults are among the population groups that use health services the most^54^. Aging is associated with increased prevalence of diseases and disabilities. For this reason, it is a phase of life in which the use of health services tends to increase^56^.

Socioeconomic characteristics can influence the patterns of use of health services. Persons with better socioeconomic conditions can obtain more easily health care services than poorer individuals. This social inequality in the access tends to be greater in countries with a private health system, in which persons have to pay for health care and insurance plans or out-of-pocket, than in countries with universal system^13^. In the United States, a country with predominantly private health system, the older adults belonging to the highest quintile have twice the chance to consult a doctor compared to the older adults of the lowest quintile. In France, which has a universal system, such difference is not seen^2^.

Education can also exert an important influence on the access to and use of health services. In addition to the strong association with income, persons with higher education tend to have greater ease in recognizing a health need and seek the care service^55,57^.

Despite the reduction of social inequalities with increasing age, these inequalities tend to persist, even to a lesser degree, and can influence the access to health services among the older adults. Moreover, the value of health insurance plans and contracts increases according to age, favoring the access of the wealthier older population^24^.

In addition to the individual characteristics, social inequalities in the access to and use of health services are also an expression of the characteristics of the health system. The availability of diagnostic and therapeutic equipment and services, the geographical distribution, the mechanisms for the financing of services, and their organization represent characteristics of the system that may facilitate or hinder the access to health services^35,53,57^.

The objective of this study was to analyze the association between the socioeconomic characteristics and the access to or use of health services among older adults.

## METHODS

This is a systematic review of the literature with articles indexed in electronic bases of the American National Library of Medicine and the National Institutes of Health (PubMed), the Web of Science, and the Virtual Health Library (VHL), using references of the Latin American and Caribbean Health Sciences Literature (LILACS).

The descriptors used to select the studies have been chosen based on Health Sciences Descriptors (DeCS) and Medical Subject Headings (Mesh terms), and other relevant terms on the subject (highlighted in italics): (“schooling attainment” OR “family income” OR “income” OR “Socioeconomic position” OR “*Socioeconomic level*” OR “*Economic level*” OR “*Assets index*” OR “Poverty” OR “*Deprivation*” OR “Schooling” OR “education”) and (“health services accessibility” OR “health services utilization” OR “access to health services”) and (“elderly” OR “*major adults* “ OR “*older people*” OR “aged” OR “*older adults*”). We have also examined the reference list of the selected studies.

The searches have been conducted without language and date restriction, to find all the existing articles; however we have included only articles published in Portuguese, English, and Spanish, because of the familiarity with the language. The databases were consulted in May 2015 and the articles were moved to the software Endnote X 7.2. The duplicates have been deleted.

The inclusion criteria were: (i) any observational design, not necessarily population-based; (ii) socioeconomic factors related to income and education as variables of interest in the analysis of the access to or use of health services among older adults; (iii) adjustment for confounding factors, at least for gender and age; and (iv) no selection bias (we considered as selection bias when reported by the author, or studies with losses greater than 50%). We have included articles that considered in their analysis of the access or use: public or private services, including medical appointments, specialist appointments, dental appointments, hospitalization, emergency services, and home visits. The articles that have assessed problems of access to or use of health services without specification have also been included. All types of measures have been considered. We have excluded the works regarding access to drugs and surgeries. We have also excluded studies relating the access or use with variables other than the socioeconomic ones.

We have included one article with persons aged over 45 years^25^ and three with individuals aged over 50 years^2,16,46^, as they are cohorts whose goal has been to evaluate events in the older population^2,15^ or they are cross-sectional studies that have the older adults as their main focus^25,46^.

We have read the title of the articles and deleted those unrelated to the subject of access to and use of health services. The abstracts were read and deleted as they did not meet the inclusion criteria. Articles whose titles and abstracts did not provide clarity regarding their inclusion or exclusion were kept for the next step to read them in full. We have selected the articles read in full that met the criteria. The process has been conducted by two researchers independently. Disagreements were discussed between the pair until a consensus.

We have found 5,096 articles after deleting duplicates. Of these, 4,873 were excluded after reading the titles and 162 eliminated after reading the summary. Of the 61 articles analyzed in their entirety, 33 have not met the inclusion criteria. Of these, three were descriptive, eighteen were not adjusted for gender and age, two had loss greater than 50%, six carried out a joint analysis of adults and older persons, in two the outcome was not the use or access, and two were excluded because they were available only in German or Korean. From the search in the references of the selected articles, eight were included, amounting to 36 articles selected for this review (Figure). Table 1 presents an overview of the studies.

**Figure:**
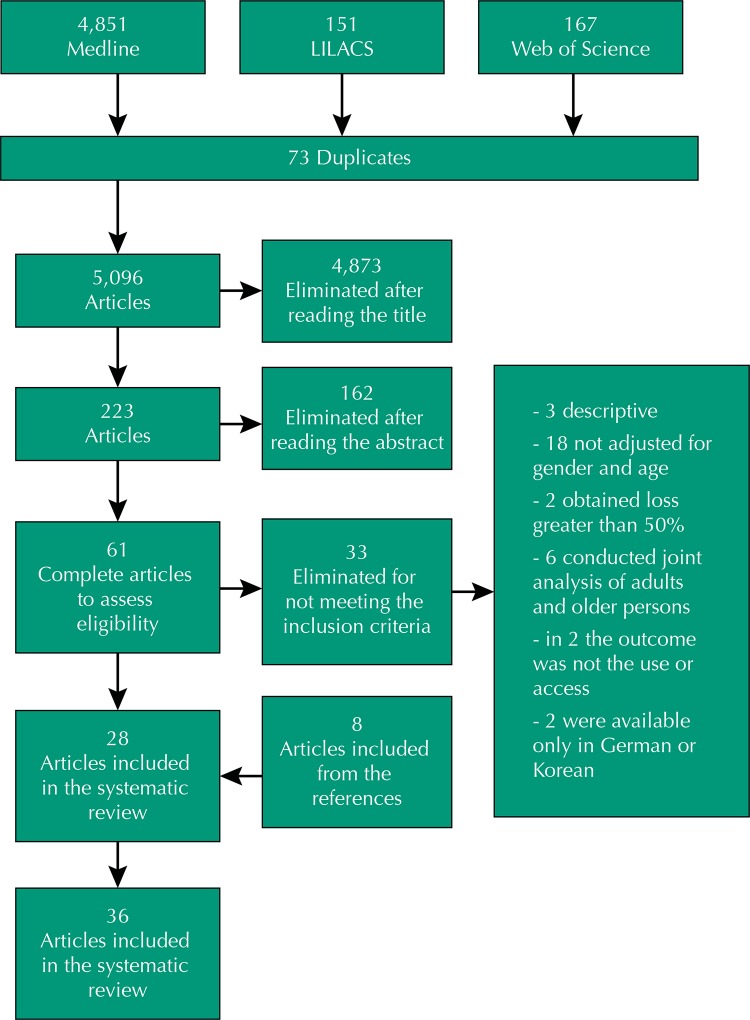
Flow diagram of the process of selection of articles in the different phases of the systematic review.

**Table 1:** Studies that have evaluated the association between socioeconomic factors and the access to or use of health services among older adults.

Author (year)	Country	Sample	Design/Age group (year)	Outcome(s)	Recall period	Downs and Black
Allan et al.^1^ (2011)	Canada	3,424	Cross-sectional/ ≥ 65	Number of MA; nights in Hospital; HV	12 months	17
Allin et al.^2^ (2009)	Austria Belgium France Denmark Greece Germany Italy Netherlands Spain Sweden Switzerland USA	1,828 3,626 1,585 2,937 2,909 2,636 2,473 2,836 2,300 2,972 932 18,148	Cross-sectional/ ≥ 50	MA, DA, and number of MA	12 months	13
Araújo et al.^4^ (2009)	Brazil	597	Cross-sectional/ ≥ 60	DA	12 months	17
Auchincloss et al.^5^ (2001)	USA	12,341	Cross-sectional/ ≥ 65	Problem in the access	12 months	17
Barros et al.^6^ (2011)	Brazil	1,518	Cross-sectional/ ≥ 60	Use of health services, hospitalization, DA	Use of services: 2 weeks; hospitalization: 12 months; DA: 1 year	17
Bazargan et al.^7^ (1998)	USA	1,114	Cross-sectional/ ≥ 62	MA, number of hospitalization, and emergency use	6 months	16
Blay et al.^8^ (2008)	Brazil	6,061	Cross-sectional/ ≥ 60	Outpatient appointment and nights of hospitalization and hospitalization	Outpatient appointment: 6 months; hospitalization: 12 months	16
Blazer et al.^9^ (1995)	USA	4,162	Cross-sectional/ ≥ 65	Have a professional reference/service; MA; nights of hospitalization	1 year	17
Blustein and Weiss^10^ (1998)	USA	7,255	Cross-sectional/ ≥ 65	Specialist appointment	1992	14
Borges-Yanez and Gomez-Dantes^11^ (1998)	Mexico	4,628	Cross-sectional/ ≥ 60	Use of health services	15 days	12
Dansky et al.^12^ (1998)	USA	6,034	Cross-sectional/ ≥ 65	MA, nights of hospitalization, and HV	1992	16
Elwell-Sutton et al.^16^ (2013)	China	30,499	Cohort/ ≥ 50	Concentration index and horizontal inequity index in the use of health services	MA: 14 days; hospitalization: 6 months	15
Fernandez-Mayoralas et al.^18^ (2000)	Spain	3,475	Cross-sectional/ ≥ 65	MA, hospitalization, and emergency use	MA: 15 days; DA: 3 months; emergency hospitalization: 1 year	15
Fitzpatrick et al.^20^ (2004)	USA	4,889	Cohort/ ≥ 65	Problems of access	1 year	12
Freedman et al.^21^ (2004)	USA	4,613	Cross-sectional/ ≥ 65	HV	12 months	12
Jiang et al.^25^ (2013)	China	2,093	Cross-sectional/ ≥ 45	Outpatient appointment and hospitalization	12 months	11
Lima-Costa et al.^28^ (2003)	Brazil	19,068	Cross-sectional/ ≥ 65	*Per capita* household income	number of MC hospitalization - last 12 months	15
Lima-costa et al.^29^ (2003)	Brazil	1,074	Cross-sectional/ ≥ 65	Total household income	12 months	17
Louvison et al.^30^ (2008)	Brazil	2,146	Cross-sectional/ ≥ 60	Outpatient appointment	4 months	16
Lupi-Pegurier et al.^31^ (2011)	France	9,233	Cross-sectional/ ≥ 60	DA	12 months	16
Matos et al.^34^ (2004)	Brazil	28,943	Cross-sectional/ ≥ 60	Time elapsed after the last DA	-	16
Murata et al.^36^ (2010)	Japan	15,302	Cross-sectional/ ≥ 65	Delay care	1 year	15
Österberg et al.^40^ (1998)	Sweden	1,778	Cross-sectional/ ≥ 65	No visit to the dentist	1 year and 5 years	16
Park^42^ (2014)	Korea	4,040	Cross-sectional/ ≥ 65	Number of MA	24 months	16
Pinheiro and Travassos^44^ (1999)	Brazil	738	Cross-sectional/ ≥ 60	Use of health services	3 months	17
Salinas et al.^46^ (2010)	Mexico	15,186	Cross-sectional/ ≥ 50	Number of MA and hospitalization	1 year	16
Sanchez-Garcia et al.^47^ (2007)	Mexico	698	Cross-sectional/ ≥ 60	DA	12 months	15
Sibbritt et al.^48^ (2010)	Australia	9,387	Cohort/ 70–75	DA	1 year	15
Strain^49^ (1990)	Canada	705	Cross-sectional/ ≥ 60	Number of MA	1 year	12
Suominen-Taipale et al.^50^ (2004)	Norway and Finland	7,919 1,283	Cross-sectional/ 65–74	MA	12 months	16
Thumé et al.^52^ (2010)	Brazil	1,713	Cross-sectional/ ≥ 60	HV	3 months	17
Wallace and Gutierrez^60^ (2005)	Brazil Chile Mexico Uruguay	2,143 1,301 1,247 1,450	Cross-sectional/ ≥ 60	No MA	12 months	14
Wan and Odell^61^ (1981)	USA	1,182	Cross-sectional/ ≥ 60	MA, DA hospitalization, and number of hospitalization	1 year	14
Wolinsky et al.^64^ (1984)	USA	15,899	Cross-sectional/ ≥ 60	Number of MA and nights of hospitalization	1 year	15
Wolinsky et al.^63^ (1983)	USA	401	Cross-sectional/ ≥ 65	MA, DA, and hospitalization	1 year	13
Wolinsky et al.^65^ (1991)	USA	5,151	Cross-sectional/ ≥ 70	MA, nights of hospitalization, and HV	1 year	15

DA: dental appointment; MA: medical appointment; HV: home visit

Selected articles have been evaluated and scored according to the methodological criteria proposed by Downs and Black^15^. The original version consists of twenty-seven items. This article has used the adapted version with seventeen items, considering the questions relevant to observational studies: 1) Is the hypothesis/objective of the study clearly defined?; 2) Are the main outcomes measured clearly described in the introduction or methods?; 3) Are the characteristics of the individuals clearly described?; 4) Is the distribution of the main confounding factors on the subject to be compared clearly described?; 5) Are the main findings of the study described?; 6) Does the study provide estimates of random variability of the data for the main outcomes (measures of variability)?; 7) Are the characteristics of the patients who were monitoring losses/losses/refusals described?; 8) Are the values of p described “accurately” rather than, for example, p < 0.05, except for p < 0.001?; 9) Are the subjects invited to participate in the research representative of the population from which they were recruited?; 10) If any of the results of the study was based on “data dredging”, is it clearly done?; 11) Are the statistical tests suitable to evaluate the main outcomes?; 12) Is the main outcome measured using accurate criteria/equipment (valid and replicable)?; 13) Were the study participants recruited in the same time period?; 14) Were the groups to be compared obtained from the same population?; 15) Are the confounding adjustments appropriate in the analysis from where the main findings were obtained?; 16) Are the monitoring losses taken into account?; 17) Does the study have enough power to detect an important clinical effect when the value of the probability for the difference due to chance is less than 5%? The question addressed receive one point for “yes” and zero for “no”, except question 4 (0 = “no”, 1 = “partially”, and 2 = “yes”), resulting in a score from zero to eighteen points.

Data extraction has been performed using a spreadsheet prepared by the authors considering the information: author, year, location, sample, objective, design, outcome(s), exposure, recall period considered in the report of access to or use of health services, measure of confidence interval and effect, adjustments made, score using the method of Downs and Black^15^, and if the article used a theoretical model to base the hierarchical analysis of the data^59^, if this approach was carried out.

The descriptive synthesis of the selected articles has been carried out according to their general characteristics, related to the information obtained by the spreadsheet. The data were discussed under the context of the health system of the countries where the studies were conducted.

Based on the measure of effect, the results of the studies were classified as ‘pro-rich’ effect (greater access to or use of health services associated with higher income and/or higher education), ‘pro-poor’ effect (greater access to or use of health services associated with lower income and/or lower education), and ‘no difference’ (the results showed no association between income and/or education and access to or use of health services).

## RESULTS

The studies are dated from 1981 to 2014; three are from the 1980s, nine from the 1990s, and 24 from the 2000s, covering a wide range of countries (36 articles in 23 countries). Three studies have been conducted in more than one country^2,50,61^. Most have been conducted in developed countries^1,2,4,7,9,10,12,18,20,21,31,36,40,42,48-50,61,64-65^ (n = 21); of these, 11 have specifically studied the United States^5,7,9,10,12,20,21,61,63-65^. Of the studies conducted in developing countries^4,6,8,11,16,24,27-29,34,44,46,47,52,60^ (n = 15), approximately two-thirds (n = 9) have specifically studied Brazil^4,6,8,28,29,30,34,44,52^.

Thirty-three studies have used cross-sectional design^1,2,4-12,18,21,24,28-31,34,36,40,42,44,46,47,49,50,52,60,61,63-65^, and three are of the cohort type^16,20,48^. The score of the studies according to the method of Downs and Black ranged from 11 to 17. Among the criteria of Downs and Black, the most frequently found limitations are: absence of description of losses and refusals, little clarity in the description of the confounding factors, no presentation of estimates of random variability of the data, and no presentation of the exact values of p.

The studies have worked with large samples: from 401^63^ to 30,499^16^. The age of the older adults has also varied. Fourteen articles have studied older persons aged over 60 years and other 14 articles have studied persons aged over 65 years, representing the majority of the studies selected. An article has examined older persons aged over 62 years^7^, another from 65 to 74 years^50^, one from 70 to 75 years^48^, and one 70 years and over^65^. We have included in this review one article with persons aged over 45 years^25^ and three with individuals aged over 50 years^2,16,46^.

Most of the publications have studied the use of health services; of these, 16 have studied more than one outcome. Of the health services investigated in the association with socioeconomic aspects, medical appointment has been studied by 16 studies^1,2,7,9,12,18,28,42,46,49,50,60,61,63-65^, 15 have studied hospitalization^1,6-9,12,18,25,28,29,46,61,63-65^ and one third of the publications (n = 12) has studied the use of dental appointments^2,4,6,18,28,31,34,40,47,48,61,63^. Two studies^7,18^ have evaluated the use of emergency services, five the home care^1,12,21,52,65^, two specialist appointments^10,50^, three outpatient appointments^8,25,30^, and four the use of any health service^6,11,16,44^.

Two articles have as outcome problems of access (yes/no)^5,20^ and one^36^ has used ‘delay care’ (delay or interruption of the search for the health service or care) as outcome. Lima-Costa^29^ has studied the main complaint or dissatisfaction when seeking medical attention.

The recall period adopted in the interviews has shown differences, ranging from two weeks to five years. Of the works that have addressed the use of dental, hospitalization, and specialist appointments, eight^2,4,6,31,47,48,61,63^, 14^1,6,8,9,12,18,24,28,29,46,61,63-65^, and two^10,50^ have used 12 months, respectively. For home visit, one article has used three months^52^, and the other has considered 12 months. For medical appointments, 14 studies have considered 12 months before the interview^1,2,7,9,12,28,46,49,50,60,61,63-65^, one has considered 15 days^18^, and one has considered 24 months^42^.

In relation to socioeconomic exposure, 21 of the studies have evaluated income and education at the same time in the model^1,2,4,5,8-10,21,24,30,31,34,40,42,44,46,47,49,52,60,64^, nine have analyzed only education^6,7,11,12,18,48,50,61,65^, and six only income^16,20,28,29,36,63^. Eleven have used total household income^1,2,5,9,10,20,21,29,31,49,52^, six *per capita* household income^16,25,28,34,36,44^, five individual income^30,40,42,64,63^, two the wealth index^2,60^, and one the economic level according to the Brazilian Association of Research Companies (ABEP)^4^. Salinas et al.^46^ have used the self-reported economic situation (excellent/very good/good, average, bad) and Sanchez-Garcia et al.^47^ have analyzed paid work, dichotomously, as exposure.

Thirty studies have investigated education level in association with the use of health services. This variable has been analyzed by most studies according to the education level in categories (16 studies). One study has considered literacy (yes, no) and two have considered if the persons have attended school, dichotomously. Eleven articles have used the variable of years of study, being five of them categorically, five continuously, and one dichotomously (more and less than six years of study). Wallace et al.^60^ have considered only the education of the head of the family.

Of the 36 selected publications, 17 have used the hierarchical model in the analysis^1,4,7,9,18,30,31,40,42,46,48,49,52,61,63-65^. Of these, 12 have adopted the behavioral theoretical model of Andersen^1,7,9,18,30,42,46,49,61,63-65^, and one has used the theoretical model proposed by the Project of Development of Methodology for Evaluating the Performance of the Brazilian Health System (PROADESS)^4^. The variables used in the adjustment have included from demographic variables and health need to behavioral variables.

Brazil (n = 9) and United States (n = 11) have been the countries with the largest number of publications in this review and those which presented a higher number of studies with association between higher income and education and use of medical appointments (30% and 60%, respectively), showing a pro-rich effect. Similar trend has been observed in studies conducted in Mexico^47,60^, Chile^60^, and Uruguay^60^. In European countries, we have found the same direction in effect between these variables in Austria, Greece, Germany, Belgium, Denmark, Italy, Switzerland, and Sweden^2^ (Table 2).

**Table 2:** Measure of effect of socioeconomic characteristics on the use of health services according to the country of study.

Outcome	Measure of effect	Country of study^reference^
Medical appointment	Pro-rich	Germany^2^, Austria^2^, Belgium^2^, Brazil^6,28,30,44^, Chile^60^, China^15^, Denmark^2^, Italy^2^, USA^2,7,12,64,65^, Greece^2^, Mexico^47,60^, Norway^50^, Sweden^2^, Switzerland^2^, Uruguay^60^
	No difference	Brazil^8,60^, Canada^1,49^, China^25^, Korea^42^, Spain^2,18^, USA^9,61,63^, Finland^50^, France^2^, Netherlands^2^, Mexico^11,46^
	Pro-poor	-
	No data	Australia, Japan
Hospitalization	Pro-rich	China^25^, Spain^18^, USA^55^
	No difference	Brazil^42^, Canada^49^, China^20^, USA^18,22,29,36^, Mexico^46^
	Pro-poor	Brazil^8,29^, USA^7,61^
	No data	Germany, Australia, Austria, Belgium, Chile, Korea, Denmark, Finland, France, Greece, Netherlands, Italy, Japan, Norway, Sweden, Switzerland, Uruguay
Dental appointment	Pro-rich	Germany^2^, Australia^48^, Austria^2^, Belgium^2^, Brazil^4,6,28,34^, Denmark^2^, Spain^2,18^, USA^2,61,63^, France^2^, Greece^2^, Netherlands^2^, Italy^2^, Mexico^47^, Sweden^2,40^, Switzerland^2^
	No difference	-
	Pro-poor	-
	No data	Canada, Chile, China, Korea, Finland, Japan, Norway, Uruguay
Emergency	Pro-rich	-
	No difference	Spain^18^, USA^7,63^
	Pro-poor	-
	No data	Germany, Australia, Austria, Belgium, Brazil, Canada, Chile, China, Korea, Denmark, Finland, France, Greece, Netherlands, Italy, Japan, Mexico, Norway, Sweden, Switzerland, Uruguay
Home visit	Pro-rich	USA^21^
	No difference	USA^12,65^
	Pro-poor	Brazil^52^, Canada^1^
	No data	Germany, Australia, Austria, Belgium, Chile, China, Korea, Denmark, Finland, France, Greece, Netherlands, Italy, Japan, Mexico, Norway, Sweden, Switzerland, Uruguay
Problems of access	Pro-rich	USA^5,20^
	No difference	-
	Pro-poor	-
	No data	Germany, Australia, Austria, Belgium, Brazil, Canada, Chile, China, Korea, Denmark, Finland, France, Greece, Netherlands, Italy, Japan, Mexico, Norway, Sweden, Switzerland, Uruguay
Specialist appointment	Pro-rich	USA^10^, Finland^50^, Norway^50^
	No difference	-
	Pro-poor	-
	No data	Germany, Australia, Austria, Belgium, Brazil, Canada, Chile, China, Korea, Denmark, France, Greece, Netherlands, Italy, Japan, Mexico, Sweden, Switzerland, Uruguay

Pro-rich: results that have favored greater use by persons with higher income or education.

Pro-poor: results that have favored greater use by persons with lower income or education.

No difference: income or education has shown no effect in relation to the use of services.

No data: lack of data considering the countries of the studies included in the review.

Income and education have shown no effect on hospitalization in eight studies and four have found a pro-poor effect. The three studies identified^7,18,63^ have shown no association between socioeconomic factors and emergency use. The use of home care in Brazil and Canada was higher in low-income persons and the use of dental appointment in all studies has been associated with higher income and mainly higher education, even in European countries.

Regarding specialist appointment, two studies^10,50^ have identified pro-rich effect in Norway, Finland, and USA.

Problems of access have been associated with the highest income and education in two studies^5,20^, both conducted in the United States (Table 2). Persons with lower income and education presented a higher chance to report problems in the access, compared to individuals with higher income and education^5,20^. In Japan, low-income citizens presented a higher chance to postpone or interrupt their search for health care (delay care)^36^.

## DISCUSSION

The association between socioeconomic factors and the access to or use of health services has varied according to the countries and type of service used. Inequality in the access to medical appointments has been identified in developing countries and in some developed countries. Increased use of dental appointments in the older population with better socioeconomic conditions has been identified in all countries. The association of socioeconomic factors with hospitalization and emergency use has been little evident: 11 studies have shown equal access^7,18,20,22,29,36,42,44,,46,63^. A pro-poor effect has been presented on the use of home visit. The use of this type of care was higher among the richest and most educated only in the United States^21^.

Socioeconomic inequalities in the access and use are related to individual characteristics, which affect the need and search for health services by the individual. They are also associated with contextual variables, especially in relation to the characteristics and form of organization of the health system, which can reinforce or hinder social inequalities in the access to health services^53,57^. It is essential to discuss the findings of this systematic review considering the context of the health systems of each country under study.

Brazil and United States are the countries that have presented the highest number of articles and most studies have pointed inequalities in the use of medical appointments^2,6,7,12,28,39,44,64,65^. These countries present similarities in the form of access to services for the older population, despite differences in the predominant funding of the health systems (Brazil – universal public system; United States – private system) and coverage of public services. In the United States, the older adults aged 65 years and over are covered by public insurance, Medicare, which helps in the reduction of individual spending, but it does not cover all medical expenses^37^. Thus, the citizen can be covered by Medicare or private insurance. In Brazil, the older adults can use the public system (Unified Health System – SUS), which faces challenges in ensuring universal access, or have health insurance or private plan^41^. This conformation of the health system could allow greater access to those who can pay for the service or health plan. This increases the iniquities in the use of health services and justifies the findings.

A study of Fitzpatrick et al.^20^ has shown that 22.3% of the older population in the United States have reported financial costs as an important barrier in the access to medical appointments, followed by transport problems (21%); the problems of access have been more frequent in lower-income individuals. Lima-Costa^27^ has shown that having health insurance is associated with the largest number of visits to the doctor (four or more) in the last 12 months in the Brazilian older population.

Allin et al.^2^ have shown greater difference in the use of medical appointments by the older population in Austria, Germany, and Sweden, where the use is greater by persons of higher income or education. The health system is a compulsory social insurance in these countries. In the other countries of universal system, such as Canada^1,49^ France^2^, Spain^2,18^, with the exception of Brazil and Italy, the studies have found no association, suggesting a better performance of the system in these countries in reducing inequalities in the access to health services.

A similar result has been found in a study with the adult population in developed countries^13^. We have observed less inequality in the countries with universal system and greater pro-rich inequality in the United States and Mexico in the use of health services, two countries without universal coverage of the population.

Two studies have found increased number of specialist appointments in persons with higher income and education^10,50^. Van Doorslaer et al.^13^ have found the same in all the countries of the Organization for Economic Cooperation and Development (OECD) in relation to the adult population, with the exception of England. Despite the different characteristics of the system between the countries, wealthier adults are more likely to consult a specialist than the poor and, in most countries, more often. This may be a reflection of the difficulty of articulation between the primary and secondary level of the health system, restricting the access to the specialist. In this way, wealthy persons can consult a specialist using the private system. Authors^50^ suggest that persons with higher education are able to persuade the general practitioner to forward them even when the complaint could be cared for at the primary level.

There has been little evidence of inequality in the use of inpatient services. Studies performed in the United States^7,38^ and Brazil^8,28^ have identified greater use in the poorest socioeconomic groups. This result can be due to the severity with which social groups seek hospital services. Poor individuals may have further complications or comorbidities as they have less access to preventive services, increasing the need for hospitalization, compared to rich individuals^3^. Wang et al.^62^ have observed an increase in the likelihood of hospitalization, according to the number of chronic conditions, and public funding of the service has been associated with greater use by poorer individuals.

Significant differences in the likelihood of visits to the dentist have been observed in developed countries, suggesting greater use in the older population with better socioeconomic conditions. The same has been found by Tennestedt^51^ in the United States. According to the study, persons with higher education have reported higher number of visits to the dentist in the last year. In Brazil, older individuals with higher education and income showed higher prevalence of recent use of dental service^19^. Systematic review studies prepared by Holm-Pedersen et al.^23^ have also found disparities in the use of dental appointments in relation to income and have pointed out the costs for dental treatment as an important barrier for the access mainly for lower-income persons in Denmark, Germany, Sweden, Norway, and United Kingdom. This may be due to the fact that most public health systems do not cover dental services or cover them partially, offered mostly by private insurers or obtained out-of-pocket.

The use of home visits in Brazil and Canada has shown inequality in favor of the poorest, and there has been a pro-rich inequality in the United States. The greater use by poorer and less educated persons probably happens because of policies with a focus on equity. A similar result has been found by a systematic review prepared by Goodridge et al.^22^ According to the review, persons with low income and lower education tend to be more prone to receive health care at home than persons with better socioeconomic level in Canada.

Health systems, including public health programs, are in themselves an important social determinant. By extending public services, the health care system can ensure equal treatment for all social groups and, consequently, combat existing inequalities in the health conditions generated by social inequities, exercising a compensatory mechanism^57,a^.

The effectiveness of policies and strategies to reduce inequalities in health has been presented in studies^17,26^. A comparison between the distributions of the prevalence of hypertension shows a social gradient in the United States and the absence of this pattern in Canada, probably a result of the universal health coverage and policies directed to socially vulnerable segments in this country^26^.

Of the studies that have used hierarchical model in the analysis (n = 17), 11 have used the theoretical model of Andersen, and one has adopted the model developed by PROADESS. The Behavioral Model of Andersen is one of the pioneers and has been developed to evaluate and understand the behavior of the use of health services by individuals^57^. It covers predisposing factors, enabling factors, and health need. The model proposed by PROADESS establishes a chain of hierarchical determinants. These determinants influence, distally or proximally, the use of services, which include individual characteristics, health problems, and self-perception of health^4^.

Most of the reviewed studies have evaluated the use of health services as outcome. Two have evaluated problems of access and one has assessed the demand satisfied. Many studies adopt the use as a proxy for access to health services. However, some authors argue that access is not equivalent to the simple use of the health service^38,39,55^. The use can be understood as the evidence that access has been reached. However, rates of use do not allow the determination of the degree to which the services were not used, although they have been necessary^38^. In the use of health services, the populations who were unable to obtain medical care (lack of access) and those that did not need health services, are considered in the same category – no use of health services. We need other ways to measure access to define more clearly the extent to which the demand is being satisfied or not.

The joint evaluation of the studies shows contrasts in the thematic approach, such as the recall period of the use of the services and the measurement of the variables of education and income, implying different methods of analysis. This can influence the measure of the effect found.

The way of measuring the outcome can also have different implications, making it difficult to compare the situations studied. Some studies have evaluated the use of inpatient services dichotomously (yes; no). Other studies have used nights of hospitalization, which may reflect the complexity of the problem and the level of health of the persons. This is because it is expected that disadvantaged persons will have worse health conditions, who may require more nights of hospitalization^3,32^.

The analysis of the association between socioeconomic factors and the use of health services among older adults has some complications. The first is the difficulty of measuring the socioeconomic status in the older population. Among the persons who are retired, income and occupation status lose their meaning, making the older adults more similar in relation to that variable, which dilutes the measure of the effect^2^. Another difficult is the selection of survivors^45^. The poorest populations present a higher morbidity and mortality rate than the average^33^. In this way, the survivors tend to have better socioeconomic conditions and are healthier than those who have died, which makes the population less heterogeneous and reduces the measure of the effect.

This systematic review presents limitations, such as the absence of a third researcher for the final decision of the articles to be included in this systematic review. The selection of articles only in Portuguese, English, and Spanish can also be a limitation of the study; however, we think this is a minor effect, as only two works have not been included because they have been published in another language.

The findings of this review have shown lower use of health services and problems of access by older persons with lower income and education, varying to a greater or lesser extent according to the country and type of service used. This fact points to inequalities in the access. Dental services have shown the greatest inequality in the use. Universal health systems have shown the greatest potential to reduce inequalities in the use of services, being a reference for countries wishing to strengthen their focus on equity.

## Footnotes

a Portal DSS Brasil. O setor saúde na redução das desigualdades em saúde. Rio de Janeiro: Centro de Estudos, Políticas e Informações sobre Determinantes Sociais da Saúde; 2011 [cited 2015 Jul 2]. Available from: http://cmdss2011.org/site/2011/08/o-papel-do-setor-saude-na-reducao-das-desigualdades-de-saude/
